# Correspondence to comment on: Impact of type 2 diabetes on complications after primary breast cancer surgery: Danish population-based cohort study

**DOI:** 10.1093/bjs/znae127

**Published:** 2024-05-27

**Authors:** Kasper Kjærgaard, Jannik Wheler, Looket Dihge, Peer Christiansen, Signe Borgquist, Deirdre Cronin-Fenton

**Affiliations:** Department of Clinical Epidemiology, Department of Clinical Medicine, Aarhus University Hospital, Aarhus University, Aarhus, Denmark; Department of Clinical Epidemiology, Department of Clinical Medicine, Aarhus University Hospital, Aarhus University, Aarhus, Denmark; Department of Plastic and Reconstructive Surgery, Department of Clinical Sciences, Surgery, Lund University, Skåne University Hospital, Lund/Malmø, Sweden; Department of Plastic and Breast Surgery, Aarhus University Hospital, Aarhus, Denmark; Department of Oncology, Aarhus University Hospital, Aarhus, Denmark; Department of Clinical Epidemiology, Department of Clinical Medicine, Aarhus University Hospital, Aarhus University, Aarhus, Denmark

*Dear Editor*

We thank Jatin Naidu for the interest in our paper^1^. We acknowledge the limitations within the study regarding residual confounding from BMI and smoking status. Neither of these variables are systematically recorded in Danish registries, precluding their inclusion in our analyses. We acknowledge these limitations in our manuscript. However, as outlined in the published manuscript, the multivariable regression analyses did adjust for age.

We incorporated information on type 2 diabetes (T2D) via clinical diagnoses of T2D (based on International Classification of Disease codes E10* to E14*, G63.2, H36.0*, N08.3 and O24* [except O24.4] in the Danish National Patient Registry) and prescription use of T2D-directed medications registered in the Danish National Patient Registry. Nonetheless, as we outlined in the published manuscript, we had no information on preclinical T2D, which might have impacted our findings with a potential misclassification of the T2D diagnosis. Still, preclinical T2D would have biased our effect estimates towards the null, so seems unlikely to explain our observed increased risk of post-surgical complications after breast cancer surgery among patients with T2D.

We fully concede the need for further analyses and encourage future studies to adequately control for potential confounding from BMI and smoking. Such knowledge would help guide the development of preventive strategies. We note, however, that, in Denmark, preoperative prophylactic antibiotics are seldom prescribed, and only for specific indications. T2D with or without other concurrently co-morbidity is not within the scope of these indications. Despite these limitations, we consider our findings valid and of high relevance to surgical oncologists and healthcare professionals who care for women with breast cancer and T2D.
